# Lean Perspectives in an Organizational Change in a Scientific Direction of an Italian Research Institute: Experience of the Cancer Institute of Bari

**DOI:** 10.3390/ijerph20010239

**Published:** 2022-12-23

**Authors:** Daniele La Forgia, Gaetano Paparella, Rahel Signorile, Francesca Arezzo, Maria Colomba Comes, Gennaro Cormio, Antonella Daniele, Annarita Fanizzi, Agnese Maria Fioretti, Gianluca Gatta, Miria Lafranceschina, Alessandro Rizzo, Gian Maria Zaccaria, Angelo Rosa, Raffaella Massafra

**Affiliations:** 1 I.R.C.C.S. Istituto Tumori “Giovanni Paolo II”, Viale Orazio Flacco 65, 70124 Bari, Italy; 2 Department of Interdisciplinary Medicine, University of Bari, 70124 Bari, Italy; 3 Department of Precision Medicine, University of Campania Luigi Vanvitelli, 80131 Naples, Italy; 4 Department of Management, Finance and Technology, LUM University, 70010 Casamassima, Italy

**Keywords:** lean healthcare, lean management, lean office

## Abstract

Lean management is a relatively new organizational vision transferred from the automotive industry to the healthcare and administrative sector based on analyzing a production process to emphasize value and reduce waste. This approach is particularly interesting in a historical moment of cuts and scarcity of economic resources and could represent a low-cost organizational solution in many production companies. In this work, we analyzed the presentation and the initial management of current ministerial research projects up to the approval by the Scientific Directorate of an Italian research institute. Furthermore, the initial mode in 2021 (“as is”) and the potential mode (“to be”) according to a Lean model are studied, according to the current barriers highlighted by the final users of the process and carrying out some perspective analyses with some reference indicators.

## 1. Introduction

As the world becomes ever more competitive due to globalization in every aspect of life and given the unintended yet unavoidable consequences of COVID-19, which revealed the limitations of health systems worldwide, an overall performance optimization in every field becomes necessary [1], especially health. Authors such as Akmal and colleagues [2] in their research underline how healthcare organizations (HOs) constantly face challenges aimed at delivering more efficient and effective care to patients [3,4]. Internal inefficiencies may involve delays or insufficient maintenance [5,6,7]; thus, effective health management strategies that rationalize resources and influence clinical choices are crucial today [8]. The term “Lean Healthcare” reflects the necessity to be more attentive toward the efficiency and the final satisfaction of patients, which may be achieved by avoiding delays, errors, or inappropriate and redundant procedures [9]. Among different possible and suitable approaches [9] in healthcare is Six Sigma and Lean Thinking. Six Sigma is known for process improvement [10] and has been used in combination with Lean, according to Rathi et al. [11], to remove waste and reduce variability. Lean Thinking methodology aims to identify and remove “waste” to maximize the value of the whole system and is strictly related to the concept of supply chain management [2]. Lean methodology is used in different settings and scenarios to maximize capacity and efficiency, with its multi-faceted approach originating from the Japanese automobile industry. A multinational company incorporated its principles to respond effectively to market demands by eliminating waste and inefficiencies by applying five Lean principles [12].

Several literature reviews have been published describing the potential applications and limitations of Lean Healthcare [3,13,14,15,16]. For example, de Souza [14] tried to evaluate the research status quo by proposing an evaluation based on the differentiation between theoretical papers and case studies. Poksinska [15] portrayed how Lean has been implemented in the healthcare sector, presenting barriers, challenges, outcomes, and general mechanisms involved in the application. Holden [16] produced a critical review that implemented an analytical framework to focus on emergency care settings. Other literature reviews evaluate Lean applications in specific contexts or compare various process improvement approaches.

Despite the presence of numerous documents, we have not found a study that applied the principles of Lean specifically to the processes of evaluation and management of research projects within a scientific research institute. For these reasons, and also considering the important cultural and economic leverage that good research management produces in these contexts, we decided to investigate the potential results that the Lean methodology could allow.

In this work, the Lean methodology is analyzed within structural processes in an Italian Institute of Research (IRCCS). IRCCSs are public and private Italian hospitals that pursue research in the biomedical field and the organization and management of health services, performing high-specialty hospitalization and treatment. IRCCS research represents an investment lever involving different professional figures—both clinical and administrative—who direct many activities and finance them through numerous competitive calls.

Although this funding is essential to carry out most of their activities, each IRCCS has different criteria when selecting and approving projects submitted by various research groups. The selection is only sometimes based on objective, uniform, measurable, and unchangeable criteria, creating a series of inhomogeneities within the programming and practical management issues in research groups and the Scientific and Administrative Directorate staff. A validated and standardized procedure for allocating research funds based on objective assessments and a cost–benefit analysis could positively impact the optimization of this process, with a greater emphasis on value, reducing waste, and non-merit-based subjective assessments. Therefore, this work aims to optimize the presentation and management process of a research project within an IRCCS to verify the possible implementations from a Lean perspective.

## 2. Materials and Methods

**Figure 1:**
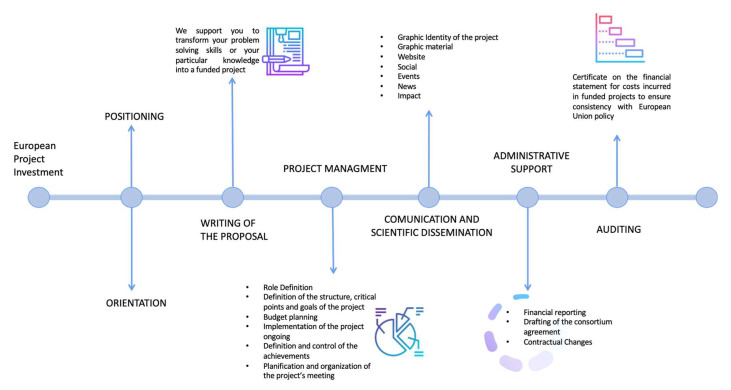
Main stages of presentation and management of a European project within a research institute.

In our elaboration regarding the Istituto Tumori “Giovanni Paolo II” Clinical Research Cancer Center of Bari, Italy, relating to the organization present in 2021, the presentation and management map of a research project was drawn up by identifying the sub-processes, the people involved, and the inputs and outputs, as can be seen in Figure 1. Furthermore, the time taken for each activity, both actual and waiting between one process and the next, was also investigated to calculate the minimum and maximum duration of the overall process in minutes, hours, and days to separate the time by value from expectations. The analysis was carried out starting from a macro perspective and gradually reaching a more detailed level.

In the elaboration concerning the “Giovanni Paolo II” Cancer Institute in Bari, the analysis was conducted starting from a macro perspective and then progressively arriving at a more detailed level. During the mapping, the team members used paper material to take notes and identify the importance, timing, and lead time of the sub-process activities and the sub-processes. Those principles are correlated to the creation of a Value Stream Map (VSM), the starting point of any Lean implementation [17], including in healthcare practices [18,19,20]. VSM allows us to have a clear picture of the trend of the value flow before making any Lean implementations and noting any activities that do not bring value and only generate waste.

The Value Stream Map is derived from the upstream collection of all the detailed information on the current state of each process (defined as “as is”): by consulting the leader for each step, the team members involved in the processes reported all the necessary information describing the process, its development, how many people are involved and how much work awaits in each area. We analyzed the following steps currently (“as is”) in force in our Institute for the approval of projects related to the Current Research calls of the Ministry of Health in Italy:

(1) Acknowledgment of the announcement and authorization for internal disclosure by the Scientific Director: this disclosure takes place through the library via emails sent to all Institute staff potentially involved in the research.
(2) Sending of tenders to the administrative staff: the tender is communicated to the administrative offices of the Institute for information to activate the consequential procedures.
(3) Writing of projects by individual researchers: the researchers interested in the call begin independently to write research projects based on specific skills by informing the director of their operating unit.
(4) Sharing of projects with other research partners: researchers look for internal and/or external partners to the Institute with whom to share their project to enhance it.
(5) Definition of the projects between Principal Investigator and partners: the projects are defined in detail and validated by all the researchers involved, who also define roles, budget to request, and internal management.
(6) Evaluation of the projects by the Scientific Director: evaluation of the individual projects with verification of feasibility and consistency with the research objectives.
(7) Validation by the General Manager: the Scientific Director proposes the projects to be financed and establishes their budget. The Institute’s Strategic Management acknowledges them and, if it agrees, validates the choice.
(8) Peer review: before final approval, the Scientific Director hears the technical opinion of the Institute’s external reviewers on the projects presented and which are intended to be approved.
(9) Communication of the results in official form through a resolution published on the Institute’s website.

In a Lean Team, effective communication is essential; therefore, leaders of each operation need to describe the sequence of their duties and how they communicate with upstream and downstream process operators. By doing so, team members can draw on the VSM on how the information flow and material are carried out throughout various processes.

In the third phase, the team focuses on the time required for each phase of the process. We verified with the operation managers the actual time of carrying out the value activity and the waiting time for each step by calculating the minimum and maximum time for each procedure based on the history.

**Figure 2:**
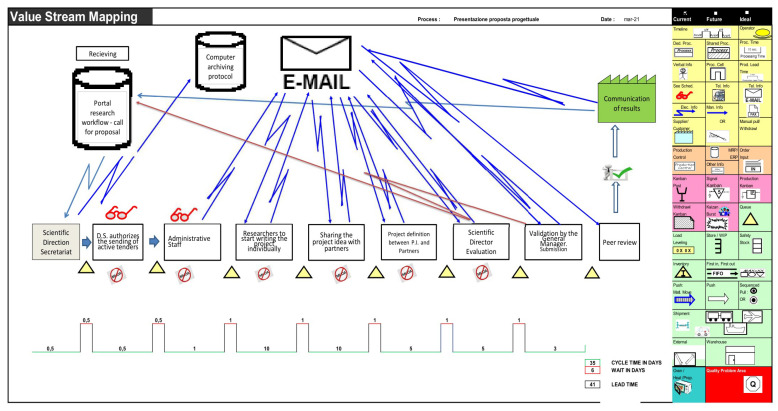
Actual state “AS IS”.

After collecting those data, the Lean Team was able to draw the Value Stream Map of the process, shown in Figure 2.

The activities of different professional figures were evaluated: researchers, doctors, technicians, nurses, administrators, and engineers belonging to different operating units inside and outside the Institute. In addition, an anonymous multi-choice survey was filled out by full-time researchers, doctors, and biologists of the operational units involved in the research to identify the problems and any resistance to change. The questionnaire, consisting of 31 multiple choices and 1 final open question, was sent through the mailing list of our library to about 300 addresses.

The purpose of the questionnaire was to identify the main barriers, strengths, and weaknesses in the presentation and management path of the Institute’s internal research projects.

It was also specified that the questionnaire, filled in anonymously, was not aimed at evaluating individual or operating units’ performances but only the problems and difficulties inherent in the path mentioned above from the perspective of researchers representing the end users of the process.

In multiple-choice questions specific to the different departments and processes, users had the following answer options:

(a) Completely satisfied;
(b) Very satisfied;
(c) Satisfied;
(d) Unsatisfied;
(e) Completely unsatisfied/missing.

Answers were divided in binary form into satisfied users (answers a, b, and c) and dissatisfied users (answers d and e).

**Figure 3:**
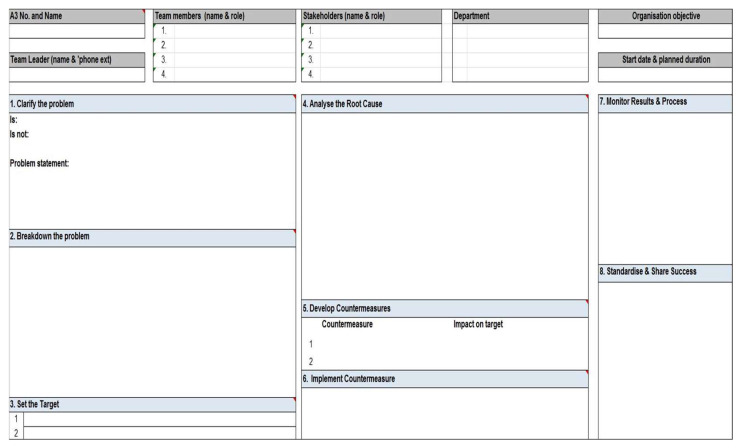
A3 template for immediate visual identification of process issues, root causes, and possible solutions.

To explore a problem within a beneficial process is the A3 tool that, through its description and analysis of the current situation and root causes, proposes objectives and countermeasures through an implementation plan and monitoring of the results. An example is shown in Figure 3.

Once the processes were analyzed, and waste was reported, team members identified areas where an improvement was possible, and based on the existing VSM, a predictive VSM could be drawn. Finally, an action plan was planned, including information such as future actions, the person responsible for the development, the deadline for the implementation, and the state of the action. To create the future VSM, the Lean Team followed seven different questions:

- What does the researcher need, and when;
- How often do performances need to be tested according to the researcher’s needs;
- Which steps create value and which waste;
- How can interruptions be reduced during the workflow;
- How can interruptions be controlled;
- Is it possible to balance the workload and the different activities;
- What process improvements will be needed.

## 3. Results

**Figure 4:**
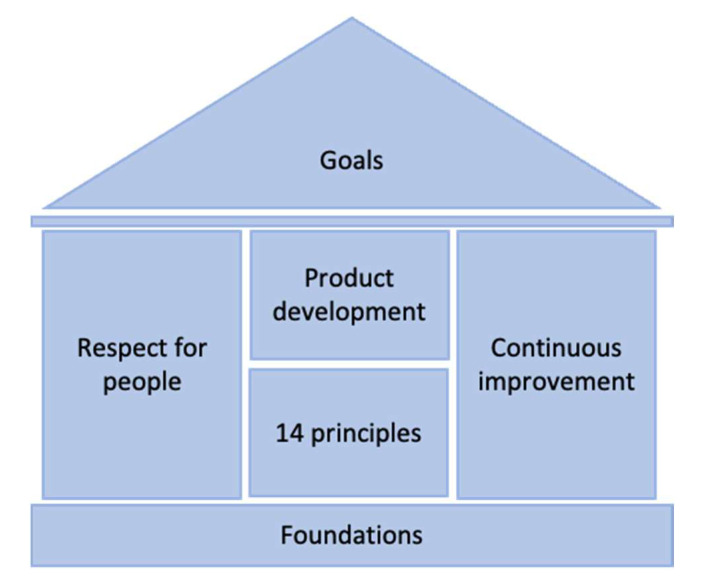
Lean Thinking house.

In the first analysis, we considered the first 50 questionnaires received and highlighted the points of most significant dissatisfaction. Once the barriers were identified, we focused on solutions and changes needed in offices from a Lean perspective based on the following criteria:

1. Creation of a Lean Team that will teach team members strategies and benefits of a Lean Office, starting with the involvement of the office manager; once the consensus by the Responsible Manager is acquired, the most motivated and involved members will be selected and informed about the fundamental information of the method and the teamwork will be activated according to the principles of Lean Thinking House—Figure 4.
2. Selection of a product or service, focusing on the sector of greatest interest.
3. Mapping the process in detail through a VSM of the current state of the process.
4. Brainstorming with detailed analysis of the possible changes following Lean principles.
5. Production of a VSM for the future state based on the consideration obtained from the previous point.
6. Support and verification of the changes in a Kaizen perspective through the involvement and monitoring of the Lean group of the Institute.

In total, 50 people answered the questionnaire. The reports pointed out that the greatest concern was the valorization of merit, as 42 and 40 out of 50 revealed little to no valorization in scientific and overall merit, respectively. Other critical aspects concerned the internal training received to draw up and write a research paper which was deemed as “lacking” by 39/50 of researchers, the assistance for the drafting and sending (38/50), the interaction between internal and external scientific groups (37 and 36 out of 50, respectively), missions (27/50), the time and administrative procedures needed to acquire in research projects personnel (35/50), equipment (32/50), and materials and consumer goods (32/50). We summarized these data in the following Table 1.

Barriers were grouped and summarized by the following topics and were associated with the departments involved to understand the principal causes:

- Meritocracy;
- Research assistance;
- Coordination of research;
- Administrative procedures for the acquisition of goods and services.

Through the analysis of the answers in the questionnaires, it was possible to identify the following problems:

1. The Scientific Directorate does not have an office responsible for selecting calls/grants but almost always uses only the information received from the Ministry of Health or the Puglia Region.
2. Once received, calls are almost always sent to all researchers without any selection based on the competencies and research field of the individual researchers. In this way, the administrative offices of the Scientific Directorate save time. However, the work of researchers is aggravated because they need to investigate by reading notices not relevant to them.
3. The researcher is left alone in formulating the project idea and compiling all the annexes (often complicated) to be sent for the project proposal submission.
4. The strategic direction of the Institute in the field of research is unclear to some researchers.
5. The choice of possible project partners is often made by the proposing researcher without a shared strategy with the Scientific Directorate and the Directorate General.
6. The project proposal, which is not shared with the technical areas (feasibility study), is submitted to the Scientific Director only when the idea is defined, which often entails the return of sums not properly spent.
7. Lack of dedicated administrative practices in research groups and for interactions between these, the Scientific Directorate, and other administrative offices.
8. The Institute lacks a native English-speaking translator for the numerous linguistic revisions of scientific articles sent to journals.
9. In the research project management phase, there is a lack of standardized processes, internal office procedures, and procedures relating to relations with other administrative offices.

Following the identification of the waste, a new VSM was drafted, answering the seven questions previously reported: those answers led to the request for the creation of a Grant Office to manage the process, starting from the selection of calls to propose up to the administrative management and for a dialogue between researchers and the institute administration.

Once “MUDA” (process information, physical environment, and behavior) were reported, opportunities for improvement were hypothesized, implementing an action plan to solve the detected problems and seizing the opportunities for improvement.

After analyzing the processes, Lean Team members determined that the current process could be improved. Based on the current Value Stream Map, the team can start brainstorming to realize an ideal future VSM. An action plan is thus created, including the following data: actions to be taken, identification of the person responsible for its development, deadline for the implementation, and status of the action (planned, under development, or executed).

A color is assigned to each state of the action so that better visual control can be obtained and it is clearer on which tasks more focus is needed.

Following the procedure described, the Lean Team, based on the following seven questions, created the VSM of the future state (“to be”):

(1) What does the researcher need and when?

In this case study, the creation of a Grant Office is required to manage the entire process, ranging from the selection of calls for proposals to the administrative management of the same.

It also emerged from the questionnaires the need for a greater comparison of researchers with the administrative part of the offices of the Institute and, in particular, a special office of the Scientific Direction to guide the researcher (who has medical skills) in the management and definition of issues, often administrative–managerial.

(2) How often should we check our performance according to the needs of the researcher?

The Scientific Director will review the Grant Office for each process and will also be checked by the researcher whenever a change is made to the standardized procedure that will be defined for implementing the processes.

(3) Which steps create value and which waste?

According to the current VSM, shown in Figure 2, it can be said that the management of the analyzed activity without any specific referent certainly generates “waste”, which was also identified in the disproportionate use of email. Therefore, the Grant Office should monitor project management to avoid generating unverified versions.

(4) How can we make the workflow work with less disruption?

Due to the lack of control over the sequence of activities and the time required, keeping track of all office activities is not easy. The team then suggests using the Kanban system: this will allow one to efficiently plan tasks in real time and control and optimize pending work. Interruptions along processes can be reduced by using software applications in some sub-processes. For example, all materials can be shared in an IT platform that provides continuous updating under the supervision of the Grant Office. In this way, all the material relating to the call can be managed, compiled, and integrated remotely by all those involved in the presentation of the project idea, and the moments of confrontation in the presence would be reduced to those essential.

(5) How do we control work between breaks?

Every process in the office is carried out following push logic; there is no rule to control each activity’s priority, and the Scientific Director manages all relevant information. Thus, it would be more appropriate to implement pull logic to manage the activity under consideration, leaving the Scientific Director the possibility to define the priority of the actions to be taken. In addition, it is considered necessary to implement a system based on procedures (currently absent) that allows for standardizing the entire process (including project management).

(6) Is it possible to balance the workload and/or the different activities?

Standardizing the process steps will balance the workload. It will be possible to reduce the waiting time for corrections that the researcher may have to make, the lead time for the creation of the final project may be relatively reduced, and the quality of the materials produced will benefit as a result.

(7) What process improvements will be needed?

Analyzing the current state, the Scientific Director is the one who usually assigns, indistinctly, the calls received by the Ministry or other institutions to researchers. This step takes place by an email sent by the administrative offices. However, a massive sending of emails generates a process of overexposure that leads over time to a lack of communication between the Scientific Directorate and researchers. As a solution, implementing the “Grant Office” is proposed, which reduces the total lead time of the office and makes the process of submission of research projects more efficient.

Based on the analysis carried out, a modification of the process defined as a possible future state (“to be”) is therefore proposed with the following steps that replace the current state of the process (“as is”):

(1) Creation of a Grant Office with the task of evaluating calls from research portals, selecting, and managing them;
(2) Sending of research notices by email by specialty to individual researchers, avoiding indiscriminate sending;
(3) Preliminary meetings with the researchers to define the project idea;
(4) Presentation of the proposal to the research partners;
(5) Executive meeting and final definition of the project, budget, and roles;
(6) Submission and validation by the General Manager;
(7) Receipt and communication of results.

**Figure 5:**
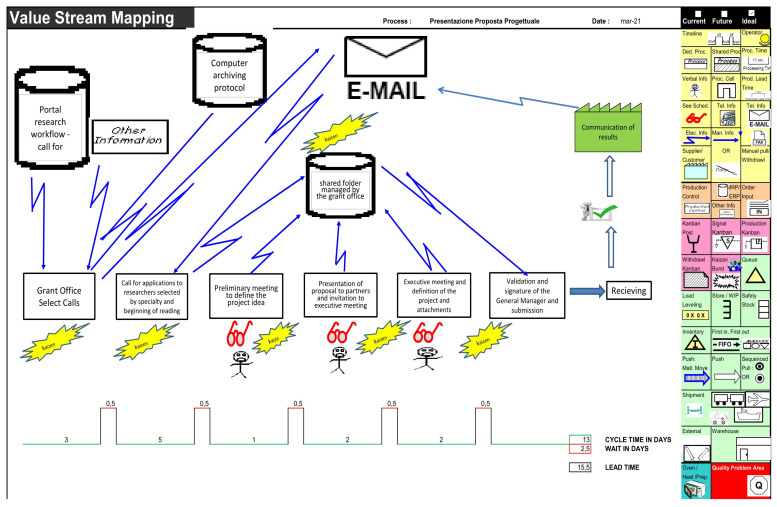
Future state (“to be”).

All the processes to improve times and communication, summarized in the VSM in Figure 5, envisage constant IT interaction between the researcher, partner, and Scientific Management via email and a shared electronic folder.

The Grant Office will be reviewed by the Scientific Director for each process as well as the researcher every time a change in the standardized procedure will be defined. According to the current VSM, the absence of a specific figure for managing the activity generates waste, and the disproportionate use of email was also identified.

Due to the lack of control over activity sequences and the time required to keep track of all the office’s tasks, we suggested the Kanban method. It is a computerized system that allows the planning of projects in real time and the managing and optimizing pending work. The platform also provides continuous updating under the supervision of the Grant Office in a pull perspective.

Creating a Grant Office would involve the acquisition of at least three professional figures: a language expert, a design expert, and an accounting specialist for a gross annual cost estimated at EUR 105,000.

The language expert should review the scientific texts and publications of the research groups and write and review the European Calls for Proposals. The design expert would be responsible for the analysis and drafting of research projects for competitive calls in collaboration with researchers of the Institute. At the same time, the accounting specialist should focus on the management and economic analysis of research groups.

Given the lack of administrative staff in the Scientific Directorate and the absence of staff dedicated to the practices of the research groups, we thought about the further acquisition of two administrative collaborators dedicated to following the practices of internal research groups to the Institute and interfacing with the administration, practices currently fully managed by the Principal Investigator (PI), with an additional cost estimated at EUR 60,000 gg per year.

The latter would be the link requested by the researchers with the Scientific Directorate and the administrative area and would support them by notifying them of the status and closure of the practices and their management: the acquisition of software to manage the Kanban boards for the mapping in real time of all the processes, updated from the same administrative ones, must be considered with an average annual license cost of EUR 15,000. Therefore, the annual costs of implementing our model would be EUR 180,000 annually, which a single additional project from the Institute can cover.

As this research is aimed at improving the efficiency of the process related to the submission and approval of research projects and, ultimately, at increasing the productivity and scientific quality of the Institute, two types of indicators were considered.

The first one is a progressive indicator which will be discussed at the beginning of each year to evaluate the status of the problem resolution through brainstorming and disseminating questionnaires like the one proposed in this initial study. Scores will be awarded for each issue highlighted following an evaluation grid with scores from 0 for non-resolution up to 2 for full resolution. The scores will then be summed up, and an overall indicator of the barriers will be provided. The latter will be compared with that of the previous year and with the basal (status “as is” without Grant Office): the percentage deviation of the prior year’s scores and the initial situation will be measured, and a process trend curve will be created. The second will be the final indicator: the difference in the total annual funding obtained for research by the Institute (estimated three-year increase +15%, desirable +20%) and the total impact factor produced (estimated annual increase +10%, desirable +20%) will be verified, as well as the number of projects submitted and scientific articles submitted in the three years following the establishment of a Grant Office.

## 4. Discussion

Lean thinking has been successfully applied in a wide variety of healthcare facilities leading to benefits such as quality, access, efficiency, and reduction in cancer mortality. However, to reach those potential benefits, healthcare organizations need to engage senior management, work across functional gaps, and aim for continuous improvement over time [21]. Part of this work aims to analyze the possible changes in the presentation and management of research projects in an Italian Cancer Institute, studying the potential barriers and proposing practical solutions.

One of the main difficulties found in different organizations is staff resistance to the change advocated by the Lean Office [22]. Thus, the analysis was focused on a single office activity. However, after demonstrating the usefulness of this implementation to the staff, it will be easier to experiment with such an innovative method in all the office areas of the Scientific Direction.

Results summarized in Table 2 highlight the exclusive involvement of the Scientific Directorate in two of the four principal barriers and partially in the other two. Regarding the meritocracy barrier, it emerges that the Scientific Directorate, together with the Heads of the different operative units, do not carry out a comparative assessment of individual researchers based on the projects, innovations, and impact factors within the single operative units.

Regarding the administrative processes, there is a lack of transparency in the procedures that are activated and concluded mostly without direct communication with the referent researcher. In some cases, procedures are not carried out in chronological order. Using the Kanban board on the intranet with reserved access from the administrative representatives and the researchers could be a solution in defining the steps and the status of the practice, reducing the time spent by both parties and resulting in reinvestment of useful time in active work.

The second and third barriers (assistance and coordination of research) highlighted the presence of a serious shortage of staff in the Scientific Directorate whose duties are the following:

- Maintaining relations with international, ministerial, and regional organizations.
- Monitoring, management, and reporting of clinical trials (resolutions, agreements, and economic management).
- Monitoring, management, and reporting of research projects (funds Current Research, targeted research, 5 × 1000, airc, capital account, European projects, and individual donations), currently about 60 ongoing projects.
- Procedures for the acquisition of services and supplies below the threshold.
- Technical secretariat of the Scientific Directorate (archiving, conferences, and scientific events’ organization).
- Activities related to the library, the management of the publications system, and the information obligation toward the Ministry of Health through the monitoring and managing of the Ministry’s Research Workflow system.

These methods align with a series of efforts to rationalize work tools in health practice and clinical prediction analysis [23,24,25,26,27,28,29]. For instance, to limit interruptions along processes, an IT implementation could be a solution so that all materials related to the call can be managed, filled out, and supplemented remotely by all the subjects involved, reducing direct engagement to the essential. Furthermore, it was noted that every process in the office is carried out following push logic, without any rule on how to prioritize activities, and the Scientific Director manages all the relevant information. Therefore, using pull logic to manage the activity under consideration could be a better option, leaving the Scientific Director the possibility to define actions’ priorities. In addition, deploying a system based on procedures allows the standardization of the entire process, project management included. In this way, the workload will be balanced, while the waiting time needed for correction by researchers and the lead time needed to plan the final project will be reduced. Currently, the calls received by the Ministry of Health or other institutions are proposed and sent by email to researchers without any distinction, leading to overexposure that bears to non-communication between the Scientific Directorate and researchers. Establishing a Grant Office with the previously described features could reduce the total lead time and make the research submission process more efficient, especially if followed by the addition of IT platforms.

Spreading the Lean culture could create a new awareness of a handy tool to rationalize processes and reduce waste. However, cultural barriers established within the hospitals on a business-originated methodology could pose a problem that could lead to identifying it as a practice aimed only at savings, cuts, and layoffs more than value and efficiency. Thus, Lean technical activities must be part of a comprehensive management system supported by committed leadership and an institutional culture of support [30,31].

In a Lean system, we move progressively from a centralized, top-down approach, which is rigid and repetitive, to a transformational leadership system. This system is increasingly empowering and flexible, with the senior management that inspires and involves the company’s employees, encourages desirable behaviors, and strengthens the ability of the organization to change from the perspective of value and satisfaction of users. This shift will increase employee engagement and support the organization’s bottom-up (pull) dynamics to create a culture of identification of non-value, proposed improvements, and the implementation of achievable and realistic solutions.

Considering the staff and the potential of the research groups, we estimated that this implementation could lead to a growth of at least 15–20% per year of the funding currently allocated to the Institute and that today is based almost exclusively on Current Research.

There is strong evidence that the quality of work (the appropriate working environment and the optimal way work is organized) affects psychological health, and that reducing psychological distress and sickness absenteeism increases workers’ well-being. This is particularly pronounced in emotional work (i.e., all service work that cannot be described by the physical component alone but must consider the interaction of users and their emotions), which is common in healthcare [32].

Improving healthcare and research quality and efficiency is a recurring challenge faced by health services. Internal inefficiencies, such as poor patient throughput and inappropriate resource utilization, can contribute to overcrowding and delays in care, impacting patient and staff satisfaction, patient safety, and overall quality of care [33].

Improving the efficiency and quality of care can be further enhanced by applying the digital technologies (DT) offered by Industry 4.0 [33].

Today, the lack of a Grant Office leaves researchers alone and without links led by the Scientific Directorate; this prevents, in most cases, the coordination of several research groups in internal and external teams to participate in competitive research calls with strong projects, especially in European projects and ministerial research.

Analyzing the data reported in the Annual Report of the IRCCS Istituto Tumori of Bari from 2015 to 2019 regarding the Sectoral Research Report, in which are reported the costs incurred during the year and financed by current and finalized research funds related to the related revenues, a low growth in the budget available for research activity was seen over the years, from EUR 3,043,062 in 2015 to EUR 3,415,735 in 2019.

The increased funding obtained through other competitive calls (today, the funded research takes place mostly through the funds of Current Research) would allow the acquisition of additional assets and personnel and, consequently, the implementation of a number of quality technological innovations, publications, and funding for Current Research.

Among the limitations of this study is one inherent to the reality of office work, clearly different from an industrial reality with much less defined phases and times. The overlapping skills or working methods do not allow complete mapping of all the process stages. However, staff awareness and education towards Lean principles and the ongoing improvement process should reduce this variability.

In this regard, the Kanban boards were adopted in the most problematic areas to trace the processes transparently and allow a quick response to users: an automatic system allows sending emails to users to warn of the status change of the practice or its completion.

Another limit that would significantly influence the results of applying Lean principles in a healthcare setting is the involvement of top management figures (Heads of Services, Department Directors, and Strategic Management).

These figures should be involved, motivated, and deeply convinced of the goodness of Lean management principles. Without their necessary active involvement and consent, the Lean logic would be challenging to apply systematically, remaining unsuccessful in most cases. Close collaboration between staff and top management is also required [34].

Our result forecasts must therefore be considered in the presence of a proactive and convinced management of the positive impact that streamlined logic can bring in a context such as the one studied.

Future research on these topics should be able to measure more precisely in economic terms the improvement in productivity linked to the advancement of the organization and the working climate, the better use of services, and the reduction in the process lead time. The latter is linked to reducing the system’s inefficiency (waste) by disseminating IT tools for participation and sharing. These times can be reinvested in new productivity without affecting working hours and the load of the individual employee.

The challenge proposed by Lean techniques, therefore, goes far beyond the simple optimization of processes, but also concerns a new way of thinking about work that is more efficient and attentive to the well-being and perspective of those who work. In addition, above all, this proposed technique concerns those who are end users of a process that become active and involved.

## 5. Conclusions

Our study underlines how modern approaches to change in complex systems can more easily achieve concrete and feasible results, starting from an analysis of the current state of processes, from their detailed and meticulous mapping, to highlighting the value according to the point of view of the end user in a Lean perspective with a small-step approach (Kaizen).

Our study has shown that the waste and organizational limitations of a simple administrative process of approval and management of ministerial research projects by a Scientific Directorate could lead to reduced growth opportunities and a consequent potential budget loss for a scientific institute.

A Lean approach, through process analysis and identification of barriers for end users in managing research projects in an Italian Oncology Institute, highlights low-cost end solutions that are easily implemented. This would bring a more efficient process and improvement in the overall scientific quality of the Institute in the long term.

Finally, even after overcoming application difficulties, using the Lean Office approach may bring significant advantages in terms of costs, time, and overall satisfaction of the stakeholders involved in the process. Therefore, it is a method that is desirable to extend highly motivated contexts to rationalize health resources and reduce waste in oncology research.

Our Institute started an important organizational change in the Scientific Direction in 2022, following this analysis’s findings, establishing a Grant Office. In future work, we will provide the real “to be” data of this reorganization to verify its results in the field.

## Figures and Tables

**Table 1:** Main issues reported in the barrier identification questionnaire.

Critical Issues Reported	N° of Reports/50	Percentage Ratio (%)
Failure to exploit scientific merit	42	84
Failure to exploit general merit	40	80
Internal training in project preparation	39	78
Assistance received at project submission	38	76
Lack of interaction between internal scientific groups	37	74
Lack of interaction with external scientific groups	36	72
Missions	27	54
Personnel acquisition time and mode	25	70
Equipment acquisition time and mode	32	64
Consumer goods acquisition time and mode	32	64

**Table 2:** Principal barriers and main causes found.

Barriers	Departments Involved	Causes
Meritocracy	General and Scientific Directions	Unclear and depreciating rules
Research assistance	Scientific Direction	Shortage of dedicated staff, absence of Grant Office
Research coordination	Scientific Direction	Shortage of dedicated staff, absence of Grant Office
Administrative procedures	Scientific Direction, Administrative Direction, Personal Area, Clinical Engineering	Shortage of staff, procedures not always analyzed by date of request

## Data Availability

Not applicable.
